# 2-methoxyestradiol Prevents LNCaP Tumor Development in Nude Mice: Potential Role of G_2_/M Regulatory Proteins

**DOI:** 10.4137/jcd.s2480

**Published:** 2009-06-16

**Authors:** Cara Horny, Muralimanoharan Sri Balasubashini, Krishna Komanduri, Manonmani Ganapathy, I-Tien Yeh, Rita Ghosh, Addanki P. Kumar

**Affiliations:** Department of Urology, School of Medicine, 7703 Floyd Curl Drive, University of Texas Health Science Center, San Antonio, TX 78229.

**Keywords:** prostate cancer prevention, 2-methoxyestradiol, apoptosis, PSA

## Abstract

Nontoxic naturally occurring metabolite of estrogen namely 2-methoxyestradial (2ME_2_) found in serum and urine has been shown to be antitumorigenic in various tumor models including the prostate. A recent study conducted in breast cancer cells showed growth stimulatory effect of 2ME_2_ when used at low concentrations (10–750 nM). Studies from our laboratory has demonstrated prostate tumor preventive ability of 50 mg/kg 2-ME_2_. In this study we show that concentrations of 2-ME_2_ as low as 1 µM is sufficient to inhibit proliferation and induce apoptosis in androgen responsive LNCaP cells. In addition oral administration of doses lower than 50 mg/kg prevented prostate tumor development in LNCaP xenograft model. The observed tumor growth inhibition was associated with induction of apoptosis, increased expression of Wee1 kinase and p34cdc2. In addition administration of 25 mg/kg 2-ME_2_ prevented tumor development significantly that is associated with reduction in serum PSA levels.

## Introduction

Prostate cancer is the most common cancer in men in the United States apart from non-melanoma skin cancer. The incidence is estimated to be 218,890 cases per year and prostate cancer accounts for over 27,000 deaths per year.^1^ The incidence has been increasing by approximately 1.1 percent annually since 1995.^1^–^3^ Evidence suggests that prostate cancer progresses from normal epithelium to proliferative inflammatory atrophy (PIA), to low grade prostatic intraepithelial neoplasia (LGPIN), to high grade PIN (HGPIN) that eventually progresses to the more aggressive-metastatic and clinically evident prostate cancer.^4^ Although such preneoplastic lesions have been found in young men in their twenties, the clinically detectable prostate cancer does not generally manifest itself until after age 60. In addition, the occurrence of precancerous lesions is more prevalent (~1 in 3 men) than the incidence of carcinoma (~1 in 9 men).^5^ The current available treatment options for early stage (organ-confined) prostate cancer are radical prostatectomy (RP), radiation therapy (external beam RT [EBRT], brachytherapy, or both), and active surveillance.^6^–^8^ Prostatectomy has shown to have an excellent success rate but adverse effects include urinary incontinence and impotence resulting from damage to the urinary sphincter and the penile nerves, which may significantly decrease the quality of life. Radiation therapy is comparatively effective to a radical prostatectomy but side effects also include incontinence and erectile dysfunction that significantly decrease the quality of life. For advanced disease, the treatment options are limited to androgen deprivation therapy which is administered with a palliative not a curative intent. The setbacks in prostate cancer treatment are twofold. First, there is the need for an equally effective curative treatment for organ confined prostate cancer without the heavy side effect profile in addition to preventing the advancement of the disease while preserving quality of life. Also, there is a need for a curative treatment for advanced disease. Interestingly the long latency involved in the development of clinically significant prostate cancer provides plethora of opportunities for intervention.^6^–^8^

Studies conducted in our laboratory showed a nontoxic naturally occurring metabolite of estrogen found in serum and urine, 2-methoxyestradial (2ME_2_) has the potential for preventing the development of early stage prostate tumor development in a transgenic adenocarcinoma of mouse prostate (TRAMP) model.^9^ 2ME_2_ has been shown to arrest the cell-cycle at the G_2_/M phase, bind to the colchicines-binding site of tubulin resulting in tubulin depolymerization, and to activate apoptotic signaling pathways.^10^–^16^ Recent studies from our laboratory also demonstrated that 2-ME_2_ has the potential not only to prevent early stage tumor development but also to regress established tumors in TRAMP mice through transcriptional regulation of FLIP.^17^ In contrast, low concentrations of 2-ME_2_ (10–750 nM) produced strong growth stimulatory effect in breast cancer cell lines tested.^18^ To the best of our knowledge it was not known whether low concentrations of 2-ME_2_ will inhibit or stimulate growth of prostate cancer cells *in vitro* and *in vivo*. In this study we show that low concentrations of 2-ME_2_ (1 μM) inhibited proliferation of androgen responsive LNCaP cells through induction of apoptosis that is associated with increased expression of Wee1 kinase and p34cdc2. In addition administration of low dose of 2-ME_2_ (25 mg/kg) through gavage prevented tumor development that is also associated with induction of apoptosis and necrosis. Further the observed tumor growth inhibition was also associated with reduction in the serum levels of PSA.

## Materials and Methods

### Animal experiments

Male nude athymic mice (Harlan), 6–7 weeks of age on Day 1, were fed *ad libitum* water (reverse osmosis, 1 ppm Cl) and an irradiated rodent diet (PMI NIH Rate and Mouse 07) consisting of 22.7% protein, 5% fat, 4.5% fiber and 7.5% ash. The animals were housed in static microinsulators on a 12-hour light cycle at 21–22 °C and 40%–60% humidity. PRC specifically complies with the recommendations of the Guide for Care and Use of Laboratory Animals with respect to restraint, husbandry, surgical procedures, feed and fluid regulation, and veterinary care. Each mouse was injected subcutaneously in the flank region with 1 × 10^7^ LNCaP prostatic adenocarcinoma in 0.2 mL saline. The mice were placed in treatment groups of 10 animals each and administered 2ME_2_ formulated in 3% DMA/40% HBC (N,N dimethyl acetamide/hydroxypropyl β-cyclodextran) for the duration of the study. The 2ME_2_ was administered at two dosing levels of 25 mg/kg and 75 mg/kg (oral daily schedule). Control group received the DMA/HBC vehicle. The study was terminated after 62 days of treatment when the tumor weight in the control group was 1 g. The tumor was excised from the euthanized animal, weighed and biopsied. Necropsy was conducted on all animals to determine if there were any gross organ abnormalities in response to 2ME_2_. The liver, lungs, kidneys, large intestine, prostate gland, urinary bladder and testes were excised and preserved in 10% neutral buffered formalin. Efficacy was evaluated by tumor size daily using the formula tumor weight (mg) = l × w^2^/2 (l and w denotes length and width of the tumor respectively). Animals with tumor weight of 1 g were euthanized prior to the 62 day time period. These animal experiments were conducted at Piedmont Research Center (Morrisville, NC) which is AAALAC accredited. Intervention studies in TRAMP mice were conducted at University of Texas Health Science Center, San Antonio, TX as described in.^17^

### Tumor histology

Tumors were harvested and fixed in 10% neutral buffered formalin. Tumors were paraffin embedded, sectioned, placed on slides, and stained with H & E to visualize cell nuclei and cytoplasm. The histopathological analysis included comparison of the tumors on the basis of differentiation and pleomorphism, variation in size, hyperchromasia and nuclear shape, nuclear to cytoplasmic ratio, number of mitoseis, abnormal mitoses and the extent of necrosis were the specific characteristics analyzed. Images were recorded using a light microscope.

### Cell lines and reagents

Androgen responsive LNCaP human prostate cancer cell line was grown in RPMI 1640 medium supplemented with 10% FBS as described previously.^9^,^13^ Polyclonal antibodies directed against cdc25c, pcdc25c, wee 1, pcdc2, cdc2 and cyclin B1 were purchased from Santa Cruz Biotechnology (Santa Cruz, CA). All other chemicals, including 2ME_2_ were from Sigma Aldrich, St Louis, MO.

### Cell proliferation assays

LNCaP cells were plated in 96-well plates at a density of 4,000 cells per well in triplicate. Following attachment (after 24 h), cells were treated with different concentrations of 2-ME_2_ (10, 50, 100, 250, 500, 750 and 1000 nM; 3 μM). Control cells received only the solvent (DMSO). Cell proliferation was detected after 72 h of incubation with using Cell Titer One Aqueous solution assay (Promega Corporation, Madison, WI) as described previously.^9^,^13^

### Preparation of cell extracts and western blot analysis

Exponentially growing LNCaP cells were treated at 70%–80% confluence with 1 μM 2ME_2_ for 2, 6 and 12 hour time periods. Cells were lysed following treatment. The lysed cells were passed through a 21-guage needle and centrifuged at 12,000 rpm for 20 minutes, to remove cell debris. The protein content of the extracts was determined by the Bradford Method as described before.^9^,^13^ Equal amounts of extracts were fractioned on a 10% sodium dodecyl sulfate-polyacrylamide gel and electrophoretically transferred to a nitrocellulose membrane. Bound antibody was detected by Enhanced Chemiluminescence as described previously.^9^,^13^

### Detection of apoptosis in cells and tumors

LNCaP cells were treated with a 1 μM 2ME_2_ and harvested after a two hour incubation period. The cells were trypsinized to terminate the reaction and centrifuged at 1,500 rpm for 10 minutes. The amount of apoptosis was detected using the Apo DETECT Annexin V-FITC kit (EMD Chemicals, Inc. Gibbstown, NJ). Tumor cell apoptosis was detected the TUNEL assay (Promega corporation, Madison, WI).^17^

### Determination of serum PSA

Serum levels of PSA was measured using sandwich ELISA based assay according to the manufacturer’s instructions (Cal Biotech Inc., Spring valley, CA). Briefly, binding of PSA present in the serum to PSA antibody coated plates will be detected using HRP labeled secondary antibody. Following this colorimetric based assay was used to determine the concentration of PSA. Concentration of PSA in the serum samples was determined by generating a standard curve using increasing amounts of PSA.

### Statistical analysis

Most of the cell culture experiments were repeated thrice and some were repeated four times. Data are presented as average ± s.d and significance was determined using student’s t-test. The differences between the experimental groups was considered to be significant at p < 0.05.

## Results and Discussion

**Figure 1:**
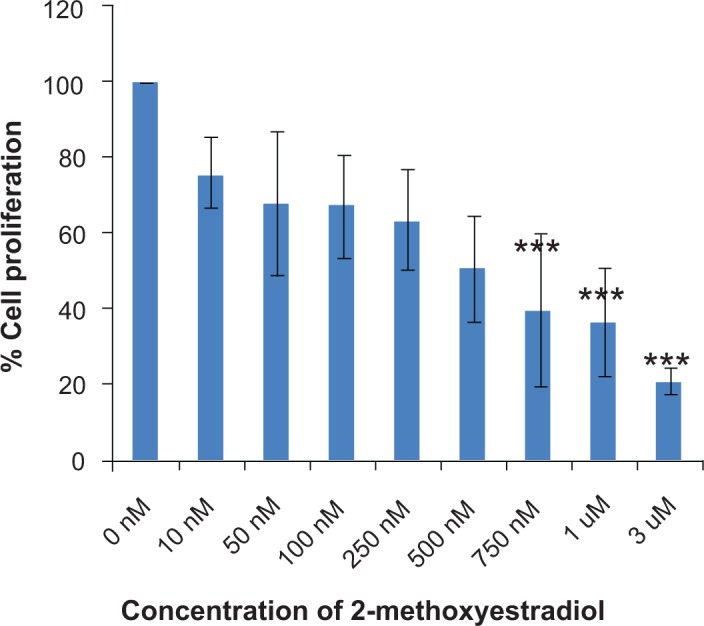
2-ME_2_ inhibits proliferation of LNCaP cells: Androgen responsive (LNCaP) cells were plated in 96-well plates as described in materials and methods and treated with indicated concentrations of either 2-ME_2_ or solvent control. Cell proliferation was determined using Cell Titer96 aqueous One solution assay at 72h and normalized to the proliferation obtained in the absence of 2-ME_2_. The data shown are an average ± sd of three replicate wells and is a representative of four independent experiments.

We examined the effect of 2ME_2_ on proliferation of androgen responsive LNCaP and androgen independent PC-3 human prostate cancer cells using the CellTiter96 Aqueous one solution assay as described earlier.^9^,^13^ Exponentially growing cells were treated with different concentrations of 2-ME_2_ and cell proliferation was measured after 72 h as described in materials and methods. As shown in Figure 1, significant inhibition of proliferation was achieved with concentrations as low as 750 nm (approximately 60% inhibition) in LNCaP cells. However 1 μM 2-ME_2_ was necessary for a similar effect in PC-3 cells (Fig. 1; data not shown). Based on these data we selected 1 μM 2-ME_2_ for subsequent experiments. These data indicate that concentration of 2-ME_2_ lower than 1 μM inhibits proliferation of prostate cancer cells. However we did not observe growth stimulatory effect that was reported in breast cancer cells at the low concentrations.^18^

**Figure 2:**
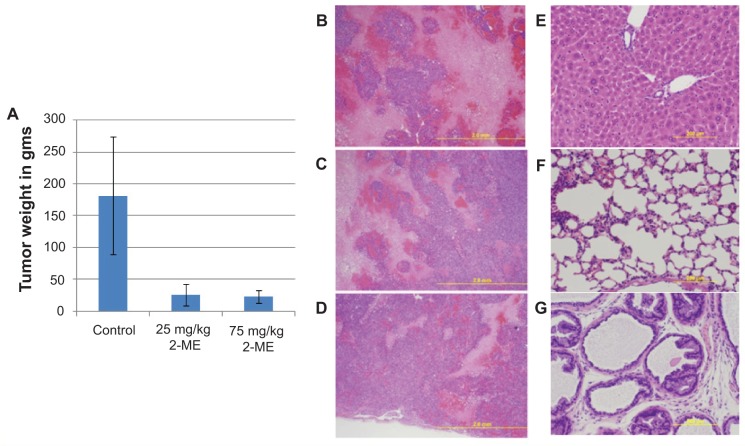
Effect of 2-ME_2_ on tumor development in nude mice. Thirty athymic male nude mice (6–7 week old) were randomized into three groups of 10 animals each and injected with LNCaP cells subcutaneously in the flank region. 2ME_2_ was administered to the mice beginning at the time of inoculation at doses consisting of 25 and 75 mg/kg on oral daily schedule until the study was terminated on day 62. Panel (**A**) shows the mean tumor weight in each of the three groups. Shown here are the histological photographs of the LNCaP tumors from the control (**B**), 25 mg/kg (**C**) and 75 mglkg (**D**) 2ME_2_ treatment groups. Histological analysis of the LNCaP tumors showed a greater confluence of necrotic area in the control group compared with the 2ME_2_ treatment groups. Photographs of the liver (**E**), lung (**F**) and prostate (**G**) of the treatment groups taken under light microscopy at 4 × magnification is also shown. No differences in control and treatment groups were observed.

As shown in Figure 2a, the mean tumor size at the end of the 62 day time period in the control group was 181.2 ± 92.9. In the 25 mg/kg and 75 mg/kg treatment groups the final mean tumor sizes were 26 ± 17.3 and 23.2 ± 10, respectively. In the 25 mg/kg treatment group only one mouse reached the 1 g termination point but the remainder of the mice survived to the experiment endpoint. However we did not observe any significant difference in the incidence of tumor development between different groups. The Body weight measurements were taken daily to evaluate the side effect profile and acute toxicity. Oral administration of 2-ME_2_ had no significant effect on body weight changes. Since LNCaP cells are known for poor tumor uptake (one of the fallbacks of the tumor implantation process using LNCaP cells), the responses characterized as complete tumor regressions were grouped as poor tumor takes and the data was discarded from calculation. These data demonstrating significant reduction of tumor size in response to 2ME_2_ oral dosing is consistent with the published data in genetically engineered mouse model.^9^ However that study used much higher dose (50 mg/kg) through dietary intervention as opposed to oral gavage using 25 mg/kg in the current study.

Histopathological evaluation of the tumor sections showed greater extent of necrosis in the control group compared to treatment group (Figs. 2b–d). Necrosis in human tumors has been associated with a poorer prognosis. There is a functional relationship in the cell to limit death by necrosis by the ability of a cell to undergo apoptosis or autophagy. If a cell fails to control its fate in inopportune circumstances by apoptosis or autophagy, necrosis ensues which produces an inflammatory response. This inflammatory response is associated with enhanced tumor growth.^19^ As demonstrated above treatment with 2ME_2_ decreases the amount of necrosis and therefore it may also curbs tumor progression associated with inflammation (Figs. 2b–d). Interestingly we did not observe any gross abnormalities in the liver, lungs, kidneys, large intestine, prostate gland urinary bladder and testes in response to 2-ME_2_ intervention (Figs. 2e–f). These data indicate that 2ME_2_ treatment is not toxic consistent with published reports.^9^,^10^,^13^,^15^,^16^

**Figure 3:**
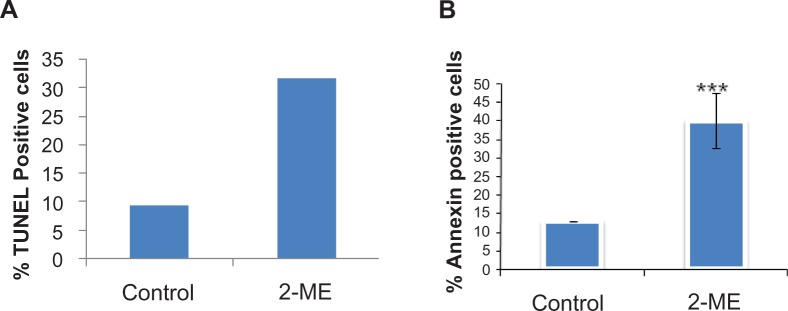
Analysis of apoptosis in tissue sections of 2ME_2_ treated LNCaP human prostatic xenograft in athymic nude mice: Apoptosis was detected using the Dead End Colorimetric TUNEL System as described in the materials and methods section using paraffin embedded tissues. Slides were then analyzed under light microscopy at 40 × magnification for change in TUNEL staining. Nuclei of treated cells showed increased brown staining, indicative of apoptosis. Nuclei of untreated cells did not show staining. The percentage of stained to unstained nuclei was counted under 40 × magnification light microscopy in the control and 2ME_2_ treatment groups from ten different fields and shown in panel A. Panel B shows apoptosis in LNCaP cells treated with 1 μM 2-ME_2_ using FITC-Annexin staining.

Apoptosis is a pathway of cell death that is induced by a tightly regulated intracellular program that plays a critical role in the maintenance of tissue homeostasis.^20^,^21^ We investigated whether the observed tumor growth inhibition is due to apoptosis using modified TUNEL assay in the tumor sections. Slides were then analyzed under light microscopy at 40 × magnification for change in TUNEL staining. Nuclei of treated cells showed increased brown staining, indicative of apoptosis (data not shown). The percentage of stained to unstained nuclei was counted under 40 × magnification light microscopy in the control and 2ME_2_ treatment groups from ten different fields and shown in Figure 3a. As shown in Figure 3a, tumor tissues from 2-ME_2_ treatment group (25 mg/kg group) showed approximately 25% apoptosis compared to less than 10% in the control group. These data indicate that 2-ME_2_ treatment prevents development of LNCaP prostate tumors in nude mice that is associated with induction of apoptosis and necrosis. Although using low doses these data showing inhibition of tumor development that is associated with apoptotic induction is consistent with the published reports in various tumor models including prostate.^9^,^10^,^13^,^15^–^17^

**Figure 4:**
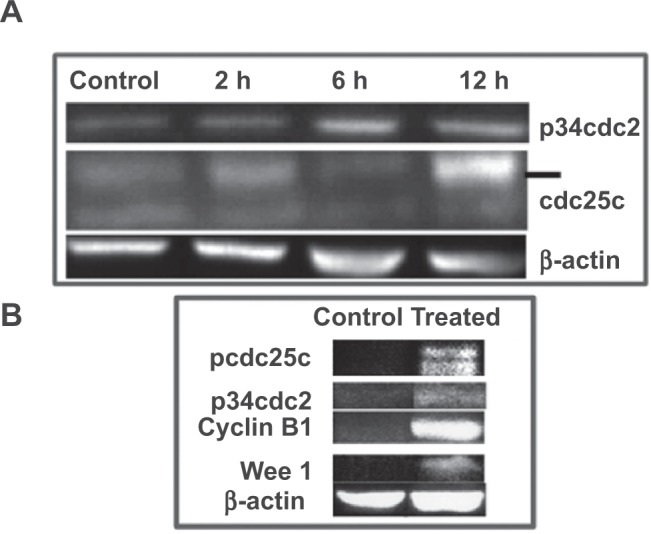
Low dose 2-ME_2_ (1 μM) treatment increases protein levels of p34cdc2 in LNCaP cells: Whole-cell extracts were prepared from LNCaP cells were used in immunoblot analysis with indicated antibodies (**A**). Whole-cell extracts were prepared separately from TRAMP prostate tumors (three individual animals on control diet or prostate tissue (dorso-lateral from three individual animals on 2-ME_2_ diet) was used in immunoblot analysis with the indicated antibodies (**B**). Bound antibody was detected by enhanced chemiluminescence using Super signal West Pico Chemiluminescent Substrate, following the manufacturer’s directions (Pierce, Rockford, IL). The blots were imaged using Syngene G Box Fredrick MD. A representative picture is shown.

Previously we have shown that 3 μM 2ME_2_ blocks cell-cycle progression particularly at the G_2_/M phase and induces apoptosis in prostate cancer cells.^13^ That study demonstrated a 14-fold increase in p21, an 8-fold increase in p34 cell division cycle 2 (cdc2) expression and an accumulation of phosphorylated cdc2 after treatment with 2ME_2_.^13^ Given the observation that both 25 or 75 mg/kg 2-ME_2_ inhibited tumor growth similarly, we investigated whether lower concentrations of 2-ME_2_ (1 μM) induce apoptosis in LNCaP cells. Treatment of LNCaP cells with 1 μM 2ME_2_ showed approximately 2–3 fold induction of apoptosis as evidenced by FITC-Annexin staining (p = 0.03; Fig. 3b). In addition at 1 μM 2ME_2_ treatment for 6–12 h, we found an increase in the protein levels of cdc25c by 3.5 fold and p34cdc2 by 2-fold (Fig. 4a). These data suggest that even lower concentrations of 2-ME_2_ inhibits growth of LNCaP cells through modulation of G_2_/M specific checkpoint and is consistent with the published reports.^11^–^14^,^22^ We also investigated whether 2-ME_2_ treatment modulates protein expression of G_2_/M specific cell cycle checkpoint proteins *in vivo*. Due to limitation in the amount of tissue availability we used prostate tissue from study conducted in TRAMP mice in western blot analysis.^17^ That study showed regression of tumors following intervention with 2-ME_2_. For this we used three independent tumor samples from each of control and 2-ME_2_ treated TRAMP mice by immunoblot analysis. As shown in Figure 4b, Wee 1, pcdc25c were undetectable in the prostate tumor from control mice but became detectable upon 2-ME_2_ treatment. Further levels of p34cdc2 and cyclin B1 was increased by approximately 2 fold in the prostate from 2-ME_2_ treated animals. These data demonstrates the modulation of G_2_/M checkpoint proteins *in vivo* that is also associated with inhibition tumor development.^17^ However the precise role of G_2_/M checkpoint proteins in inhibition of tumor development following 2-ME_2_ treatment is not known. Studies are in progress to understand their role during prostate carcinogenesis and response to 2-ME_2_.

**Figure 5:**
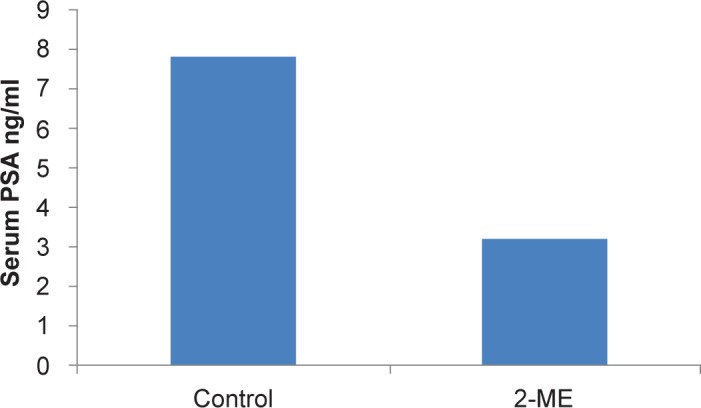
Low dose 2-ME_2_ reduces serum levels of PSA: Serum levels of PSA was measured using sandwich ELISA based assay according to the manufacturer’s instructions calorimetrically (Cal Biotech Inc., Spring valley, CA).

We also investigated whether the observed tumor growth inhibition is associated with decreased levels of serum PSA using ELISA based assay from two replicate samples from each animal. As shown in Figure 5, 2-ME_2_ treatment resulted in decreased levels of serum PSA. However the differences between control and treated groups were not statistically significant in this small sample size. However this data is consistent with published data in humans showing stabilization or decreased levels of PSA. These data is also consistent with studies conducted in hormone refractory prostate cancer patients where 2-ME_2_ administration resulted in either stabilization or decreased PSA.

In conclusion, 2-ME_2_ has enormous potential as a non-toxic chemo-preventive agent. Chemoprevention, by definition, is a pharmacologic intervention with naturally occurring or synthetic compounds that may prevent, inhibit or reverse carcinogenesis or suppress the development of invasive cancer.^23^,^24^ Prostate cancer is an ideal candidate for chemoprevention because of its high incidence, long latency period before the development of clinically significant cancer and the strong influence of environmental factors, such as food or hormones. 2-ME_2_ is an endogenous non-toxic metabolic by-product of estrogens that is present in human urine and blood. 2-ME_2_ has been shown to (i) inhibit endothelial cell proliferation implicating it in angiogenesis; (ii) inhibit the growth of different cancer cells including lung, breast, pancreatic, hepatocellular carcinoma, neuroblastoma, medulloblastoma, melanoma and gastric cancer.^10^,^12^–^18^ In addition although 2-ME_2_ is an estrogenic metabolite, it has been shown that the binding affinity of 2-ME to estrogen receptors (α and β) is very low compared to estradiol suggesting that 2-ME_2_ may not utilize signaling pathways through these receptors. Although we and several other investigators have shown that 2-ME_2_ inhibits growth of cancer cells and induces apoptosis without affecting the growth of normal cycling cells, the detailed molecular mechanism involved in mediating its growth inhibitory activity and induction of apoptosis is not clear.^10^,^12^–^18^ The mechanism of action of 2-ME_2_ is very complex and proposed to include alterations in the activities of various cell cycle regulatory proteins, transcription factor modulators (Stress activated protein kinase/Jun-amino-terminal kinase; SAPK/JNK), apoptosis regulatory proteins, regulators of cell cycle arrest, tubulin, superoxide dismutase, and binding through sex hormone binding globulin (SHBG).^9^,^17^ In addition it is known that p34 cdc2 kinase and cdc25c have been implicated in the G_2_/M progression. Activity of cdc2 kinase is regulated by phosphorylation at specific sites. Phosphorylation mediated by cdk-activating kinase (CAK) at threonine-161 residue activates it, whereas phosphorylation at tyrosine-15 mediated by Wee-1 kinase and threonine-14 and tyrosine-15 by Myt 1 makes it inactive. Alternatively, inhibition of cdc25c may lead to hyperphosphorylation of cdc2 making it inactive and blocking cells in G_2_/M. The data presented in this manuscript shows that intervention with 2-ME_2_ prevents tumor development in part through increased protein expression of Wee 1 that is associated with phosphorylation of cdc2.

Oral administration of 2-ME_2_ (75 mg/kg body weight) for 4 weeks inhibited tumor growth by about 60% with no evidence of toxicity in a breast cancer model; reduced the number of metastases in a lung tumor model by 59% and reduced tumor size in mice with angiosarcoma by 68%. We demonstrated that administration of 50 mg/kg 2-ME_2_ through diet or drinking water prevented the development of neoplastic lesions and regressed established tumors in a preclinical animal model that develops spontaneous prostate cancer.^9^,^17^ No studies have been conducted to test the efficacy of lower 2-ME_2_ in prostate cancer model. In this study we provide preliminary evidence to show that low dose 2ME_2_ inhibits prostate tumor development by attenuating cellular proliferation and inducing apoptosis. As described above, 2ME_2_ has shown to significantly decrease tumor weight on a daily oral regimen in the mouse model with no adverse effects on other organs. There was no observed organ damage or acute toxic effects from the oral administration of the medication in the mouse. Although these studies need to be confirmed using larger samples size, because of the positive effect on the androgen responsive tumors in the xenograft mouse model and low side effect profile, there is a foreseeable benefit for low dose 2-ME_2_ as a preventive agent in human patients.
